# Possible Association between Methylphenidate and Mandibular Bone Characteristics Detected by Dental Panoramic Radiograph in Children and Adolescents with ADHD

**DOI:** 10.3390/children9091276

**Published:** 2022-08-24

**Authors:** Hadas Kostiner, Lazar Kats, Nurit Kot-Limon, Eran Dolev, Sigalit Blumer

**Affiliations:** 1 Department of Pediatric Dentistry, The Maurice and Gabriela Goldschleger School of Dental Medicine, Tel Aviv University, Tel Aviv 6997801, Israel; 2 Department of Oral Pathology, Oral Medicine and Maxillofacial Radiology, The Maurice and Gabriela Goldschleger School of Dental Medicine, Tel Aviv University, Tel Aviv 6997801, Israel; 3 Department of Endodontics, Faculty of Dental Medicine, Hebrew University of Jerusalem, Jerusalem 91905, Israel; 4 Department of Oral Rehabilitation, The Maurice and Gabriela Goldschleger School of Dental Medicine, Tel Aviv University, Tel Aviv 6997801, Israel

**Keywords:** attention-deficit hyperactivity disorder, bone mass density, methylphenidate

## Abstract

Some studies have shown that children treated with psychostimulants for attention-deficit hyperactivity disorder (ADHD) have decreased bone mineral density (BMD). Mandibular cortical width (MCW) may be used as a surrogate measure for evaluating BMD. We compared the MCW measured on digital panoramic radiographs (DPR) of 38 children and adolescents with ADHD who were treated with methylphenidate for at least 12 months to the MCW of 58 children and adolescents without ADHD (control). The two groups had a similar mean age (*p* = 0.3). Mean MCW was significantly lower among children with ADHD compared to those in the control group (2.77 ± 0.33 mm vs. 3.04 ± 0.46 mm, *p* = 0.004). Additionally, each of the MCW sides were significantly smaller in the group with ADHD compared with the control group. In conclusion, treatment with methylphenidate is associated with low MCW in children and adolescents with ADHD. Analysis of MCW on DPR may help in screening children that are at risk of bone health alterations that may result in low BMD in adulthood. Dentists may be the first to identify bone health abnormalities and should be aware of their role in referring their patients to further follow-up.

## 1. Introduction

Attention-deficit hyperactivity disorder (ADHD) is the most common neurodevelopmental disorder of childhood. Among children, the pooled global prevalence of ADHD is 7.2% [1]. The literature reports a male to female ratio ranging from 2:1 to 10:1 [2,3,4,5,6]. However, it has been suggested that females with ADHD are underdiagnosed because they often have less noticeable externalizing problems [7,8]. 

Individuals with ADHD have an enduring pattern of hyperactivity–impulsivity and/or inattention that interfere with functioning and/or development [9]. Most children with ADHD are managed with a combination of behavioral and pharmacologic therapies [10]. Psychostimulants, and particularly methylphenidate, are the most commonly prescribed medications for core symptom control of ADHD in children because they improve the attention span and lower hyperactivity levels. Methylphenidate inhibits the reuptake of dopamine and norepinephrine through specific transporters in cortical and striatal areas, thereby increasing their extracellular levels in the synapse, and mediates the redistribution of vesicular monoamine transporter 2 [11]. The adverse effects of these psychostimulants, which include decreased appetite, weight loss, abdominal pain, insomnia, irritability, drowsiness, headaches, sadness, and a tendency to cry, are mainly caused by their action on the central nervous system [12,13]. 

In the last 40 years, there has been a debate whether psychostimulants—specifically methylphenidate—affect growth in children who use them. Some studies have shown that these medications may affect the child’s weight at the start of treatment [12,14,15,16] and possibly affect height in the range of 1 to 2 cm after longer-term therapy [17,18,19,20,21]. Other studies have reported that height is affected by higher doses of psychostimulants [22,23], more so in those who start treatment before puberty [24,25]. Some studies have reported that long-term treatment with methylphenidate changes the height trajectory, decreases adult height, and increases weight and body mass index [26,27,28]. 

An important factor related with growth and development is bone mineral density (BMD). The potential effects of psychostimulants on growth have led researchers to evaluate whether this drug group affects the BMD of children; however, the results of these studies are not consistent.

Lahat et al. [29] demonstrated no statistically significant difference in bone turnover markers and BMD in 10 boys aged 3–10 years who were treated with methylphenidate for 1–2 years as compared to healthy children. Poulton et al. [30] reported that that treatment of children aged 4–9 years with dexamphetamine or methylphenidate showed an association with reduced bone turnover and early fat loss. The increase in bone and other lean tissue over 3 years of continuous treatment was slower than expected for growth in height. Long-term improvement in the percentage of central fat for height was also observed. It was concluded that a relatively small decrease in the weight of children treated with psychostimulants can cause long-term changes in body composition. In another study by the same group, bone maturation over 3 years was not delayed in children treated with dexamphetamine or methylphenidate, despite the observation of decreased height and weight compared with the children’s healthy siblings [31]. Feuer et al. [32] have demonstrated that children and adolescents 8 to 20 years of age who were treated with psychostimulants had lower bone mass, mean lumbar spine BMD, bone mineral content, femoral neck bone mineral content, and total femur BMD compared with children and adolescents who were not treated with psycho stimulants. Howard et al. [28] compared total femur, femoral neck, and lumbar BMD of children and adolescents aged 8 to 17 years who were treated with ADHD medications to the BMD of matched children not receiving such medication and showed that significantly more children on ADHD medications had BMDs within the osteopenic range compared to matched subjects not receiving such medications. 

It has previously been shown that the shape and thickness of the mandibular cortex reflects bone mass loss in the body [33]. A significant correlation was reported between the cortical width of the mental foramen and BMD measured by dual-energy X-ray absorptiometry (DXA) at the lumbar spine, hip and forearm [33]. Therefore, it was suggested that dental panoramic radiograph (DPR) indices can be used to identify patients at risk for low BMD [34]. DPR is one of the most-used extraoral scans in the practice of dentistry, including pediatric dentistry. One of the important advantages of using DPR is the fact that the images obtained for diagnosis and treatment planning can be used for additional evaluations such as BMD. A commonly used index in both adults and children measures the mandibular cortical width (MCW) below the mental foramen [35,36,37]. Suboptimal peak bone mass in childhood and adolescence has been shown to predict low bone density in adulthood [38]. The controversial results, showing a possible reduction in BMD among children taking stimulants, are so far of particular concern due to the high rate of children and adolescents who are treated with psychostimulants for ADHD. Here, we compared the MCW of children and adolescents with ADHD who were treated with methylphenidate to that of a control population without ADHD. 

## 2. Materials and Methods

### 2.1. Study Setting and Participants

This cross-sectional study was carried out at the Pediatric Dentistry Department at the Goldschleger School of Dental Medicine at Tel-Aviv University, Israel. Ethical approval was granted by the institution’s Human Research Ethics Committee (approval number 115.19 dated 15 April 2019). Informed consent was given by the parents to use the data included in the children’s dental file.

Fifty children with ADHD who were treated with methylphenidate and 50 healthy children of the same age range and ethnicity who were treated in the dental clinic at the Pediatric Dentistry Department were asked to participate in the study.

The inclusion criteria for the ADHD group were children and adolescents aged 7–20 years who met the diagnostic criteria of the American Psychiatric Association for ADHD [9] and were treated with methylphenidate for a minimum of 12 months. For the control group, the inclusion criteria were children and adolescents aged 7–20 who did not have ADHD. The exclusion criteria were a history of previous treatment with psychotropic medication, medical conditions likely to impact growth (e.g., metabolic diseases), and treatment with methylphenidate for less than 12 months.

### 2.2. MCW Measurements

DPRs were taken as part of routine orthodontic examinations in Digital Imaging and Communications in Medicine (DICOM) format using a Planmeca ProMax unit (Planmeca, Helsinki, Finland). All measurements were performed by a single observer who was blinded to the participants’ diagnoses. 

**Figure 1:**
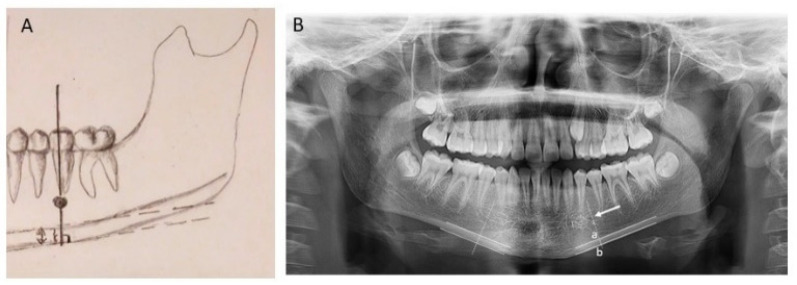
Mandibular cortical width (MCW) measurement: (**A**) A graphical representation of a MCW measurement. (**B**) A panoramic radiograph showing the measurement of mandibular cortical width. A line parallel to the long axis of the mandible and tangential to the inferior border of the mandible was drawn. A line perpendicular to this tangent and intersecting the mental foramen (dotted line) was constructed, along which the mandibular cortical width was measured. The distance between the two parallel solid lines a–b is the cortical width.

Cortex thickness of both sides of the mandible was measured on the DPRs with the RadiAnt Dicom Viewer software version 5.5 (Medixant, Poznań, Poland). As described by Paulsson et al. [36], a line was drawn on the image parallel to the long axis of the mandible and tangential to the inferior border. A line was constructed perpendicular to this tangent intersecting the inferior border of the mental foramen, along which the upper and lower delimitation points of the inferior mandibular cortex were located. Then, the MCW was measured 3 times at each side of the mandible and a mean value was calculated for each side and for both sides combined (Figure 1).

### 2.3. Statistical Analysis 

Descriptive and comparative statistics were analyzed using Statistical Software for the Social Sciences version 23 (SPSS, IBM Corp., Armonk, NY, USA). Continuous variables were compared using Student’s *t* tests or Mann–Whitney tests, as appropriate according to the type of distribution. Categorical variables were compared using chi-squared or Fisher’s exact test depending on the number of observations. A *p* value < 0.05 was considered significant. 

## 3. Results

A total of 86 children were included in the study: 38 children with ADHD and 48 children without ADHD that served as controls. No statistically significant differences in age were observed between the two groups (*p* = 0.3), but the group with ADHD had a higher percentage of males (68% vs. 48%, *p* = 0.056; Table 1).

As shown in Table 1, the MCW was significantly smaller in the ADHD group compared to the control group (2.77 ± 0.33 vs. 3.04 ± 0.46, *p* = 0.004). In addition, each of the MCW sides were significantly smaller in the ADHD group compared with the control group (Right: 2.78 ± 0.34 vs. 2.99 ± 0.48, *p* =0.02; Left: 2.76 ± 0.36 vs. 3.07 ± 0.48, *p* = 0.001).

## 4. Discussion

Over eight different methods for assessing growth have been reported in studies that evaluate growth in children with ADHD. These have included methods that are prone to artifactual distortion and low sensitivity, such as direct comparisons of mean height and frequency percentiles from standardized growth charts. The issue of method sensitivity is important when evaluating growth changes in ADHD because the reported mean height deficits have usually been small [39]. This study used DPR to evaluate the association between the use of methylphenidate for ADHD and MCW, which is a surrogate for BMD. Our results showed a statistically significant difference in MCW between children with ADHD who have been treated with methylphenidate for at least 12 months and children without ADHD, suggesting an effect of methylphenidate on BMD.

Since 1960, several studies have reported that specific changes associated with decreased BMD are visible in DPRs. These mainly refer to changes in the morphology of the inferior mandibular cortex and to altered trabecular bone architecture [33]. Usually, the cortical margin of the lower jaw is used because it is more obvious and easier to detect compared to the trabecular bone. The width of the inferior mandibular cortex is an easily measured feature that can be compared with BMD. However, it must be carefully measured in a consistent location [33].

Data from staining experiments show that the posterior surface of the ramus and the condylar and coronoid processes are the principal sites of mandible growth, while the anterior part of the mandible does not change much. In infants, the ramus is localized to the site of eruption of the primary first molar. Progressive posterior remodeling creates space for the second primary molar and then for the sequential eruption of the permanent molar teeth [40].

The area below the mental foramina is the most often studied, due to the usual lack of muscle attachment there and the fact that the distance between the mental foramen and the inferior margin of the mandibular cortical bone remains relatively stable during the lifespan, regardless of alveolar bone resorption following tooth extraction or inflammation [41]. 

A decrease in bone mass and changed morphology may be identified by the thinning and resorption of the inferior border of the mandibular cortical bone [42,43]. The most commonly used index in adults and children measures the MCW below the mental foramen [36]. Horner et al. [44] concluded that patients should be referred to DXA scanning to confirm the BMD if MCW measurements are less than 3 mm. Our results showed that most of the children in the ADHD group had an MCW lower than 3 mm.

The influence of methylphenidate on BMD may be mediated by gastrointestinal adverse effects which may change the dietary intake of calcium, thereby negatively impacting peak bone mass accrual [45]. Methylphenidate blocks catecholamine reuptake in the central nervous system, enabling higher stimulation of peripheral signaling of catecholamine receptors [46]. Additionally, norepinephrine suppresses the formation of bones and stimulates bone resorption. This effect is mediated by β2-adrenergic receptors expressed by osteoblasts [32]. Leptin also influences bone homeostasis via the β2-adrenergic pathway and may decrease appetite, as observed in individuals taking methylphenidate [47]. Therefore, the effect of methylphenidate on appetite and its potential effect on bone turnover may affect growth velocity so much so that even short-term use during the peak bone mass accrual period could detrimentally affect BMD.

Whether a smaller MCW can be exclusively attributable to treatment with methylphenidate is a subject of debate. Slower growth may also be related to associated delays in cognitive and physical brain maturation or represent a digression from the typical development pattern [48].

Diminished bone accrual during childhood and adolescence may lead to an increased risk for osteoporosis, and consequently to higher fracture risk and potentially altered fractured healing in adulthood [32]. Children and adolescents who are treated with methylphenidate and their parents may need to be educated about adequate nutritional intake and behavior modifications and receive closer follow-up. Physicians should monitor children for growth deficits so that those requiring a change in their medication regimens will be identified.

The limitations of this study include its cross-sectional design; hence, changes over time could not be evaluated. The use of medication for ADHD was self-reported; therefore, we do not have data on dosage or changes in therapy. Furthermore, the MCW of children with ADHD who are not treated with methylphenidate was not evaluated. The use of DPR for measuring MCW has some limitations, including its two-dimensional representation and observer variability. Additionally, intrinsic distortional effects may occur, which may be caused by patient positioning (which is more difficult in young children), machine motion, the region studied, and mandible morphology [49,50]. It is recommended that vertical measurements be made in regions that are anatomically oriented in the plane as the center of the image layer [51]. However, as MCW is measured approximately in this plane, and as long as the patient is carefully positioned during radiography, the distortional effects would probably have little impact on MCW measurement [42]. In the current analysis, only one observer performed the measurements, preventing the assessment of interobserver variability.

## 5. Conclusions

The present study suggests that treatment with methylphenidate is associated with low MCW in children and adolescents with ADHD. Analysis of MCW on DPR is a simple method that can help in screening children that are at risk for bone health changes that may cause low BMD in adulthood. Dentists may be the first to recognize alterations in bone health and should be aware of their role in referring their patients to further follow-up. Awareness should be raised among physicians and parents to the potential health risks of BMD associated with methylphenidate and other psychostimulants and to the importance of educating and improving the follow-up of this risk group. We suggest that dentists should conduct a bi-annual follow-up of MCW in children and adolescents who are treated with psychostimulants.

## Figures and Tables

**Table 1:** Patient’s demographic and clinical characteristics.

Variable	ADHD N = 38	Control N = 48	*p* Value *
Age (years)	12.05 ± 3.24	12.76 ± 2.88	0.3
Gender			
Male	26 (68%)	23 (48%)	0.056
Female	12 (32%)	25 (52%)	
Cortical width (mm)	2.77 ± 0.33	3.04 ± 0.46	0.004
Cortical width, left (mm)	2.76 ± 0.36	3.07 ± 0.48	0.001
Cortical width, right (mm)	2.78 ± 0.34	2.99 ± 0.48	0.02

Continuous variables presented as mean ± standard deviation and categorical variables are presented as the number of cases (percentage). * Continuous variables were compared using Student’s *t* tests or Mann–Whitney tests, as appropriate by the type of distribution. Categorical variables were compared using chi-squared or Fisher’s exact test depending on the number of observations.

## Data Availability

The data that support the findings of this study are available from the corresponding author, upon reasonable request.

## References

[1] Thomas R., Sanders S., Doust J., Beller E., Glasziou P. (2015). Prevalence of attention-deficit/hyperactivity disorder: A systematic review and meta-analysis. Pediatrics.

[2] Nøvik T.S., Hervas A., Ralston S.J., Dalsgaard S., Rodrigues Pereira R., Lorenzo M.J. (2006). Influence of gender on attention-deficit/hyperactivity disorder in Europe—ADORE. Eur. Child Adolesc. Psychiatry.

[3] Ramtekkar U.P., Reiersen A.M., Todorov A.A., Todd R.D. (2010). Sex and age differences in attention-deficit/hyperactivity disorder symptoms and diagnoses: Implications for DSM-V and ICD-11. J. Am. Acad. Child Adolesc. Psychiatry.

[4] Willcutt E.G. (2012). The prevalence of DSM-IV attention-deficit/hyperactivity disorder: A meta-analytic review. Neurotherapeutics.

[5] Arnold L.E. (1996). Sex differences in ADHD: Conference summary. J. Abnorm. Child Psychol..

[6] Gaub M., Carlson C.L. (1997). Gender differences in ADHD: A meta-analysis and critical review. J. Am. Acad. Child Adolesc. Psychiatry.

[7] Fraticelli S., Caratelli G., De Berardis D., Ducci G., Pettorruso M., Martinotti G., Di Cesare G., di Giannantonio M. (2022). Gender differences in attention deficit hyperactivity disorder: An update of the current evidence. Riv. Psichiatr..

[8] Mowlem F.D., Rosenqvist M.A., Martin J., Lichtenstein P., Asherson P., Larsson H. (2019). Sex differences in predicting ADHD clinical diagnosis and pharmacological treatment. Eur. Child Adolesc. Psychiatry.

[9] APA (2013). Diagnostic and Statistical Manual of Mental Disorders: DSM-5.

[10] Wolraich M.L., Hagan J.F., Allan C., Chan E., Davison D., Earls M., Evans S.W., Flinn S.K., Froehlich T., Frost J. (2019). Clinical Practice Guideline for the Diagnosis, Evaluation, and Treatment of Attention-Deficit/Hyperactivity Disorder in Children and Adolescents. Pediatrics.

[11] Spencer R.C., Devilbiss D.M., Berridge C.W. (2015). The cognition-enhancing effects of psychostimulants involve direct action in the prefrontal cortex. Biol. Psychiatry.

[12] Schachar R.J., Tannock R., Cunningham C., Corkum P.V. (1997). Behavioral, situational, and temporal effects of treatment of ADHD with methylphenidate. J. Am. Acad. Child Adolesc. Psychiatry.

[13] Storebo O.J., Pedersen N., Ramstad E., Kielsholm M.L., Nielsen S.S., Krogh H.B., Moreira-Maia C.R., Magnusson F.L., Holmskov M., Gerner T. (2018). Methylphenidate for attention deficit hyperactivity disorder (ADHD) in children and adolescents—Assessment of adverse events in non-randomised studies. Cochrane Database Syst. Rev..

[14] Cevikaslan A., Parlak M., Ellidag H.Y., Kulaksizoglu S.C., Yilmaz N. (2021). Effects of methylphenidate on height, weight and blood biochemistry parameters in prepubertal boys with attention deficit hyperactivity disorder: An open label prospective study. Scand. J. Child. Adolesc. Psychiatr. Psychol..

[15] Gurbuz F., Gurbuz B.B., Celik G.G., Yildirim V., Ucakturk S.A., Seydaoglu G., Ucakturk E.M., Topaloglu A.K., Yuksel B. (2016). Effects of methylphenidate on appetite and growth in children diagnosed with attention deficit and hyperactivity disorder. J. Pediatric Endocrinol. Metab..

[16] Koonrungsesomboon K., Koonrungsesomboon N. (2020). The Effects of Methylphenidate Treatment on Child Growth in Thai Children and Adolescents with Attention-Deficit/Hyperactivity Disorder. J. Child Adolesc. Psychopharmacol..

[17] Bereket A., Turan S., Karaman M.G., Haklar G., Ozbay F., Yazgan M.Y. (2005). Height, weight, IGF-I, IGFBP-3 and thyroid functions in prepubertal children with attention deficit hyperactivity disorder: Effect of methylphenidate treatment. Horm. Res..

[18] Dura-Trave T., Yoldi-Petri M.E., Gallinas-Victoriano F., Zardoya-Santos P. (2012). Effects of osmotic-release methylphenidate on height and weight in children with attention-deficit hyperactivity disorder (ADHD) following up to four years of treatment. J. Child Neurol..

[19] Kang K.D., Yun S.W., Chung U., Kim T.H., Park J.H., Park I.H., Han D.H. (2016). Effects of methylphenidate on body index and physical fitness in Korean children with attention deficit hyperactivity disorder. Hum. Psychopharmacol..

[20] Zeiner P. (1995). Body growth and cardiovascular function after extended treatment (1.75 years) with methylphenidate in boys with attention-deficit hyperactivity disorder. J. Child Adolesc. Psychopharmacol..

[21] Zhang H., Du M., Zhuang S. (2010). Impact of long-term treatment of methylphenidate on height and weight of school age children with ADHD. Neuropediatrics.

[22] Satterfield J.H., Cantwell D.P., Schell A., Blaschke T. (1979). Growth of hyperactive children treated with methylphenidate. Arch. Gen. Psychiatry.

[23] Charach A., Figueroa M., Chen S., Ickowicz A., Schachar R. (2006). Stimulant treatment over 5 years: Effects on growth. J. Am. Acad. Child Adolesc. Psychiatry.

[24] Turan S., Akay A. (2020). The effects of methylphenidate on weight, height, and body mass index in Turkish children and adolescents with ADHD. Alpha Psychiatry.

[25] Diez-Suarez A., Vallejo-Valdivielso M., Marin-Mendez J.J., de Castro-Manglano P., Soutullo C.A. (2017). Weight, Height, and Body Mass Index in Patients with Attention-Deficit/Hyperactivity Disorder Treated with Methylphenidate. J. Child Adolesc. Psychopharmacol..

[26] Greenhill L.L., Swanson J.M., Hechtman L., Waxmonsky J., Arnold L.E., Molina B.S.G., Hinshaw S.P., Jensen P.S., Abikoff H.B., Wigal T. (2020). Trajectories of Growth Associated With Long-Term Stimulant Medication in the Multimodal Treatment Study of Attention-Deficit/Hyperactivity Disorder. J. Am. Acad. Child Adolesc. Psychiatry.

[27] Swanson J.M., Arnold L.E., Molina B.S.G., Sibley M.H., Hechtman L.T., Hinshaw S.P., Abikoff H.B., Stehli A., Owens E.B., Mitchell J.T. (2017). Young adult outcomes in the follow-up of the multimodal treatment study of attention-deficit/hyperactivity disorder: Symptom persistence, source discrepancy, and height suppression. J. Child Psychol. Psychiatry.

[28] Howard J.T., Walick K.S., Rivera J.C. (2017). Preliminary Evidence of an Association Between ADHD Medications and Diminished Bone Health in Children and Adolescents. J. Pediatric Orthop..

[29] Lahat E., Weiss M., Ben-Shlomo A., Evans S., Bistritzer T. (2000). Bone mineral density and turnover in children with attention-deficit hyperactivity disorder receiving methylphenidate. J. Child Neurol..

[30] Poulton A., Briody J., McCorquodale T., Melzer E., Herrmann M., Baur L.A., Duque G. (2012). Weight loss on stimulant medication: How does it affect body composition and bone metabolism?—A prospective longitudinal study. Int. J. Pediatric Endocrinol..

[31] Poulton A.S., Bui Q., Melzer E., Evans R. (2016). Stimulant medication effects on growth and bone age in children with attention-deficit/hyperactivity disorder: A prospective cohort study. Int. Clin. Psychopharmacol..

[32] Feuer A.J., Thai A., Demmer R.T., Vogiatzi M. (2016). Association of Stimulant Medication Use With Bone Mass in Children and Adolescents with Attention-Deficit/Hyperactivity Disorder. JAMA Pediatrics.

[33] Graham J. (2015). Detecting low bone mineral density from dental radiographs: A mini-review. Clin. Cases Miner. Bone Metab..

[34] Valerio C.S., Trindade A.M., Mazzieiro E.T., Amaral T.P., Manzi F.R. (2013). Use of digital panoramic radiography as an auxiliary means of low bone mineral density detection in post-menopausal women. Dento Maxillo Facial Radiol..

[35] Calciolari E., Donos N., Park J.C., Petrie A., Mardas N. (2015). Panoramic measures for oral bone mass in detecting osteoporosis: A systematic review and meta-analysis. J. Dent. Res..

[36] Paulsson-Bjornsson L., Adams J., Bondemark L., Devlin H., Horner K., Lindh C. (2015). The impact of premature birth on the mandibular cortical bone of children. Osteoporos. Int..

[37] Apolinario A.C., Figueiredo P.T., Guimaraes A.T., Acevedo A.C., Castro L.C., Paula A.P., Paula L.M., Melo N.S., Leite A.F. (2015). Pamidronate affects the mandibular cortex of children with osteogenesis imperfecta. J. Dent. Res..

[38] Wren T.A., Kalkwarf H.J., Zemel B.S., Lappe J.M., Oberfield S., Shepherd J.A., Winer K.K., Gilsanz V. (2014). Longitudinal tracking of dual-energy X-ray absorptiometry bone measures over 6 years in children and adolescents: Persistence of low bone mass to maturity. J. Pediatrics.

[39] Spencer T., Biederman J., Wilens T. (1998). Growth deficits in children with attention deficit hyperactivity disorder. Pediatrics.

[40] Proffit W.R., Fields H.W.J., Larson B.E., Sarver D.M. (2019). Contemporary Orthodontics, 6e: South Asia Edition-E-Book.

[41] Tounta T.S. (2017). Diagnosis of osteoporosis in dental patients. J. Frailty Sarcopenia Falls.

[42] Devlin H., Horner K. (2002). Mandibular radiomorphometric indices in the diagnosis of reduced skeletal bone mineral density. Osteoporos. Int..

[43] Taguchi A., Tsuda M., Ohtsuka M., Kodama I., Sanada M., Nakamoto T., Inagaki K., Noguchi T., Kudo Y., Suei Y. (2006). Use of dental panoramic radiographs in identifying younger postmenopausal women with osteoporosis. Osteoporos. Int..

[44] Horner K., Devlin H., Harvey L. (2002). Detecting patients with low skeletal bone mass. J. Dent..

[45] Tortolani P.J., McCarthy E.F., Sponseller P.D. (2002). Bone mineral density deficiency in children. J. Am. Acad. Orthop. Surg..

[46] Faraone S.V. (2018). The pharmacology of amphetamine and methylphenidate: Relevance to the neurobiology of attention-deficit/hyperactivity disorder and other psychiatric comorbidities. Neurosci. Biobehav. Rev..

[47] Takeda S., Elefteriou F., Levasseur R., Liu X., Zhao L., Parker K.L., Armstrong D., Ducy P., Karsenty G. (2002). Leptin regulates bone formation via the sympathetic nervous system. Cell.

[48] Shaw P., Eckstrand K., Sharp W., Blumenthal J., Lerch J.P., Greenstein D., Clasen L., Evans A., Giedd J., Rapoport J.L. (2007). Attention-deficit/hyperactivity disorder is characterized by a delay in cortical maturation. Proc. Natl. Acad. Sci. USA.

[49] Nikneshan S., Sharafi M., Emadi N. (2013). Evaluation of the accuracy of linear and angular measurements on panoramic radiographs taken at different positions. Imaging Sci. Dent..

[50] Riecke B., Friedrich R.E., Schulze D., Loos C., Blessmann M., Heiland M., Wikner J. (2015). Impact of malpositioning on panoramic radiography in implant dentistry. Clin. Oral Investig..

[51] Dutra V., Susin C., da Costa N.P., Veeck E.B., Bahlis A., Fernandes Ada R. (2007). Measuring cortical thickness on panoramic radiographs: A validation study of the Mental Index. Oral Surg. Oral Med. Oral Pathol. Oral Radiol. Endod..

